# Efficacy and safety of S‐1 monotherapy in previously treated elderly patients (aged ≥75 years) with non‐small cell lung cancer: A retrospective analysis

**DOI:** 10.1111/1759-7714.13622

**Published:** 2020-08-26

**Authors:** Hisao Imai, Hiroyuki Minemura, Takayuki Kishikawa, Yutaka Yamada, Kensuke Suzuki, Yukihiro Umeda, Satoshi Wasamoto, Norimitsu Kasahara, Shinichi Ishihara, Ou Yamaguchi, Ichiro Naruse, Junji Uchino, Keita Mori, Kenya Kanazawa, Yoko Shibata, Takashi Kasai, Takayuki Kaburagi, Kyoichi Kaira, Koichi Minato

**Affiliations:** ^1^ Division of Respiratory Medicine Gunma Prefectural Cancer Center Ota Japan; ^2^ Department of Respiratory Medicine, Comprehensive Cancer Center, International Medical Center Saitama Medical University Hidaka Japan; ^3^ Department of Pulmonary Medicine Fukushima Medical University Fukushima Japan; ^4^ Division of Thoracic Oncology Tochigi Cancer Center Utsunomiya Japan; ^5^ Division of Respiratory Medicine Ibaraki Prefectural Central Hospital Kasama Japan; ^6^ Division of Internal Medicine Toyama Prefectural Central Hospital Toyama Japan; ^7^ Third Department of Internal Medicine Faculty of Medical Sciences, University of Fukui Eiheiji Japan; ^8^ Division of Respiratory Medicine Saku Central Hospital Advanced Care Center Saku Japan; ^9^ Innovative medical research center Gunma University Hospital Maebashi Japan; ^10^ Department of Internal Medicine Isesaki Municipal Hospital Isesaki Japan; ^11^ Division of Respiratory Medicine Hidaka Hospital Takasaki Japan; ^12^ Department of Pulmonary Medicine Kyoto Prefectural University of Medicine Kyoto Japan; ^13^ Clinical Research Promotion Unit Clinical Research Center, Shizuoka Cancer Center Nagaizumi Japan

**Keywords:** Advanced non‐small cell lung cancer, elderly patients, S‐1 monotherapy, subsequent‐line therapy

## Abstract

**Background:**

S‐1 monotherapy is effective and feasible for previously treated patients with advanced non‐small cell lung cancer (NSCLC). However, it is not clear whether its effectiveness and tolerability in elderly patients are equivalent to those in younger patients. Hence, this study aimed to evaluate the efficacy and feasibility of S‐1 monotherapy in elderly patients with NSCLC who had previously received other treatments.

**Methods:**

We included 96 elderly patients (aged ≥75 years) with advanced NSCLC treated with S‐1 alone as a subsequent‐line treatment at 12 medical facilities between January 2005 and March 2018 in this study. The baseline characteristics of the patients, response to S‐1 monotherapy, and adverse events (AEs) were investigated, retrospectively.

**Results:**

A total of 68 male and 28 female patients (median age, 78 [range: 75–86] years) were analyzed. In elderly patients who were treated with S‐1 monotherapy as a subsequent‐line treatment, the objective response rate, disease control rate, median progression‐free survival (PFS), and overall survival (OS) were 8.3%, 43.8%, 3.4 months, and 9.6 months, respectively. Observed AEs included anorexia, anemia, nausea, fatigue, reduced platelet count, and skin hyperpigmentation. Treatment‐related death was observed in one patient because of pneumonitis. In patients who experienced no progressive disease, subsequent‐line S‐1 alone was associated with longer PFS and OS.

**Conclusions:**

S‐1 monotherapy is effective and feasible as a subsequent‐line treatment in elderly patients who were previously treated for NSCLC, and it produces results. S‐1 monotherapy could be one of the treatment choices for elderly patients with previously treated NSCLC.

## Graphical abstract

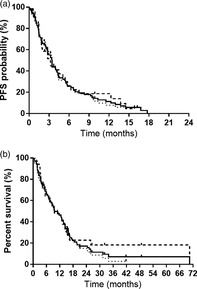

## Introduction

Non‐small cell lung cancer (NSCLC) is the second most common cancer globally and a major cause of cancer‐related death.^1^ As the elderly population continues to increase worldwide, the number of elderly patients with advanced NSCLC is rising on a global scale.^2^ The percentage of elderly individuals in Japan has increased markedly in recent years owing to the country's improved life expectancy; older individuals currently comprise more than 20% of the population. Therefore, the number of elderly patients with NSCLC in Japan is expected to rise sharply. Currently, approximately 50% of patients with NSCLC are 70 years or older,^1^ and NSCLC accounts for approximately 85% of all lung cancers among adult and aged individuals.^3^ However, the appropriate administration of chemotherapy to elderly patients remains a pressing concern. Although the incidence of malignancies among elderly individuals is rising, patients older than 75 years of age account for less than 10% of cases who enroll in the National Cancer Institute cooperative group trials; as such, elderly patients with NSCLC are underrepresented in clinical trials.^4^, ^5^ This is attributable to multiple factors, particularly older age, poor performance status (PS), insufficient social aid, and comorbidities. However, previous studies have shown that 3/4 of individuals older than 70 years of age are eager to participate in clinical trials.^6^, ^7^

Single agents such as docetaxel and vinorelbine are often administered as first‐line chemotherapeutic agents to elderly patients with advanced‐stage NSCLC in Japan. In a recent randomized phase III trial which compared pemetrexed + carboplatin therapy maintained by single‐agent pemetrexed treatment to docetaxel therapy alone in patients aged 75 years or older with advanced nonsquamous NSCLC, Okamoto *et al*. reported an objective response rate (ORR), progression‐free survival (PFS), and overall survival (OS) of 36.8%, 6.4 months, and 18.7 months, respectively, in the carboplatin plus pemetrexed combination group.^8^ However, there are still no established standard subsequent‐line treatments for elderly patients with NSCLC.

S‐1 (Taiho Pharmaceutical Co., Ltd., Tokyo, Japan) is an oral anticancer agent composed of tegafur, 5‐chloro‐2,4‐dihydroxypyridine, and potassium oxonate in a molar ratio of 1:0.4:1..^9^ Tegafur is a prodrug that is gradually converted to 5‐fluorouracil and is rapidly catabolized by dihydropyrimidine dehydrogenase in the liver. A phase III trial that compared the efficacies of S‐1 monotherapy and docetaxel monotherapy for patients with advanced NSCLC previously treated with platinum‐combination chemotherapy revealed that S‐1 was not inferior to docetaxel in terms of OS.^10^ While their study did not set an upper age limit for enrollment (the oldest patient in their S‐1 group was 85 years old), there was no detailed description of the response for patients over 75 years old; therefore, the efficacy and safety of S‐1 for patients 75 years of age and older remains unclear. Although some studies of first‐line therapy with S‐1 have been performed on a small number of elderly patients with NSCLC,^11^, ^12^, ^13^, ^14^ few have involved subsequent‐line therapies. Furthermore, most such studies were not disease‐specific, or they targeted only small subpopulations of individuals aged 75 years and older. Therefore, there remain insufficient data relevant for S‐1 monotherapy, particularly for elderly patients with NSCLC. It is also unclear whether elderly patients who received prior treatment for NSCLC should be treated with S‐1 monotherapy. Given the growing number of aging persons globally and the apparent association between age and NSCLC, clarity regarding the efficacy and feasibility of available therapeutic choices is necessary.

Hence, we retrospectively evaluated the efficacy and safety of subsequent‐line therapy of S‐1 monotherapy for elderly patients with NSCLC who had previously been treated with other agents.

## Methods

### Patients

Between January 2005 and March 2018, we reviewed the records of 96 consecutive individuals aged 75 years and older with NSCLC who had been administered S‐1 monotherapy as subsequent‐line chemotherapy at 12 medical facilities. The institutional review board at each facility approved the study protocol; the requirement for obtaining informed consent was waived owing to the retrospective study design. We retrospectively reviewed the clinical effectiveness and safety profile of S‐1 monotherapy as subsequent‐line therapy. The eligibility criteria constituted either histologically confirmed, inoperable stage III and IV NSCLC or postoperative recurrence. Prior to commencing chemotherapy, the TNM stage was assessed for each patient based on the seventh edition of the TNM staging method via a physical examination, plain chest radiography, truncal computed tomography, ^18^F‐fluorodeoxyglucose positron emission tomography or bone scintigraphy, and brain magnetic resonance imaging or computed tomography. The medical chart for each elderly patient was reviewed at each institution to obtain baseline patient characteristics as well as responses and adverse events (AEs) following subsequent‐line S1 monotherapy.

All patients were S‐1‐naïve prior to receiving subsequent‐line S‐1 monotherapy. S‐1 was administered orally, twice daily after meals, at a dose based on body surface area (<1.25 m^2^, 80 mg/day; ≥1.25 to <1.5 m^2^, 100 mg/day; and ≥1.5 m^2^, 120 mg/day) for four weeks in a six‐week cycle or two weeks in a three‐week cycle. Some patients had an irregular dosing regimen, such as two weeks in a four‐week cycle or three weeks in a five‐week cycle, as prescribed by the attending physician. The schedule and dose were modified according to the medical condition of each patient or any toxicity observed following the previous chemotherapy regimens or S‐1 cycles. Subsequent‐line S‐1 monotherapy administration continued until disease progression, development of infeasible AEs, or withdrawal of the patient's approval. If disease progression occurred after a patient had been administered S‐1 monotherapy, the patient was permitted to receive subsequent treatments beyond consultation with the attending physician.

### Treatment response evaluation

The best overall response and maximum tumor shrinkage were recorded as the tumor responses. The judgment and confirmation of therapeutic effects were performed by the attending physician. Radiographic tumor responses were assessed in accordance with the Response Evaluation Criteria in Solid Tumors, v1.1,^15^ as follows: complete response (CR), dissipation of all target lesions; partial response (PR), at least a 30% decrease in the sum of the target lesion diameters with the summed baseline diameters as a reference; progressive disease (PD), an increase of at least 20% in the sum of the target lesion diameters compared to the smallest sum during the study; and stable disease (SD), insufficient shrinkage to qualify as PR and insufficient expansion to qualify as PD. The minimum observation period from baseline was eight weeks for determining the tumor response as SD. The overall objective response rate (ORR) and disease control rate (DCR) were defined as follows: the rate of patients with CR + PR and the rate of patients with CR + PR + SD, respectively.

### Statistical analysis

Fisher's exact test was applied to analyze categorical variables. PFS was calculated from the beginning of S‐1 monotherapy until PD or death from any cause, and OS was recorded from the first day of treatment until death, or was censored on the date of the last follow‐up. The survival curves were calculated using the Kaplan–Meier method. The Cox proportional hazards regression model using the stepwise method was adjusted to identify factors associated with PFS and OS and to calculate the hazard ratios and their 95% confidence intervals (CIs). *P*‐values <0.05 were considered statistically significant for all tests. The two‐tailed significance level was also set at 0.05. AEs that were associated with S‐1 monotherapy were graded in accordance with the Common Terminology Criteria for Adverse Events (CTCAE) version 4.0. All statistical analyses were conducted using the JMP version 11.0 for Windows (SAS Institute, Cary, NC, USA).

## Results

### Patient background

Between January 2005 and March 2018, 96 elderly patients with advanced NSCLC (68 men and 28 women) with a median age of 78 (range: 75–86) years received subsequent‐line S1 monotherapy, and their characteristics are shown in Table 1. Overall, 70 of the patients ranged in age from 75 to 79 years old and 26 patients were 80 years old or older. In assessing the PS, there were 88 patients with PS 0‐1 and 8 with PS 2‐4. Moreover, Table S1 lists the chemotherapy treatments prior to S‐1 monotherapy; cytotoxic drug therapeutic regimens were the most commonly used, such therapeutic chemotherapy. In most patients with epidermal growth factor receptor *(EGFR)* mutations, EGFR tyrosine kinase inhibitors (TKIs) were used as an early treatment line. By the data cutoff date (31 August 2019), only one patient (1.0%) was still receiving (or undergoing follow‐up for) S‐1 monotherapy. The median follow‐up time was 9.1 months.

**Table 1:** Patient characteristics

Characteristic	*N* = 96	(%)
Sex		
Male	68	70.8
Female	28	29.2
Age (years)		
Median	78	
Range	75–86	
ECOG performance status score		
0	9	9.4
1	79	82.3
2	7	7.3
≥3	1	1
Smoking status		
Current or former	68	70.8
Never	28	29.2
Histology		
Adenocarcinoma	53	55.2
Squamous cell carcinoma	35	36.5
Adenosquamous cell carcinoma	2	2.1
Not otherwise specified	5	5.2
LCNEC	1	1
Treatment line		
Second	34	35.4
Third	27	28.1
Fourth	22	22.9
Fifth	8	8.3
Sixth	3	3.1
Seventh	2	2.1
Driver mutations (*EGFR, ALK*)		
*EGFR*‐mutation positive	17	17.7
*ALK*‐translocation positive	0	0
Wild‐type, negative, or unknown	79	82.3
PD‐L1 TPS		
<1%	3	3.1
1%–49%	5	5.2
≥50%	7	7.3
Unknown	81	84.4
Stage		
IIIA	16	16.7
IIIB	18	18.7
IV	51	53.1
Postoperative recurrence	11	11.5
Comorbidity		
Hypertension	22	22.9
Diabetes mellitus	19	19.8
COPD	5	5.2
Administration of S‐1 (initial dosing)		
2w1w	43	44.8
4w2w	36	37.5
2w2w	8	8.3
3w2w	5	5.2
Alternative day	4	4.2
Number of S‐1 cycles		
2w1w		
Median	3	
Range	1–16	
4w2w		
Median	2	
Range	1–10	
Reason for discontinuation of administration		
Progressive disease	73	76
Adverse events	22	22.9
Continuing administration of S‐1 at data cutoff	1	1

2w1w, two weeks of S‐1 administration followed by one week of rest; 2w2w, two weeks of S‐1 administration followed by two weeks of rest; 3w2w, three weeks of S‐1 administration followed by two weeks of rest; 4w2w, four weeks of S‐1 administration followed by two weeks of rest; ALK, anaplastic lymphoma kinase; COPD, chronic obstructive pulmonary disease; ECOG, Eastern Cooperative Oncology Group; EGFR, epidermal growth factor receptor; LCNEC, large cell neuroendocrine carcinoma; PD‐L1, programmed death‐ligand 1; TPS, tumor proportion score.

Data are expressed as n (%) unless otherwise specified (*N* = 96).

### Treatment efficacy and survival

**Figure 1:**
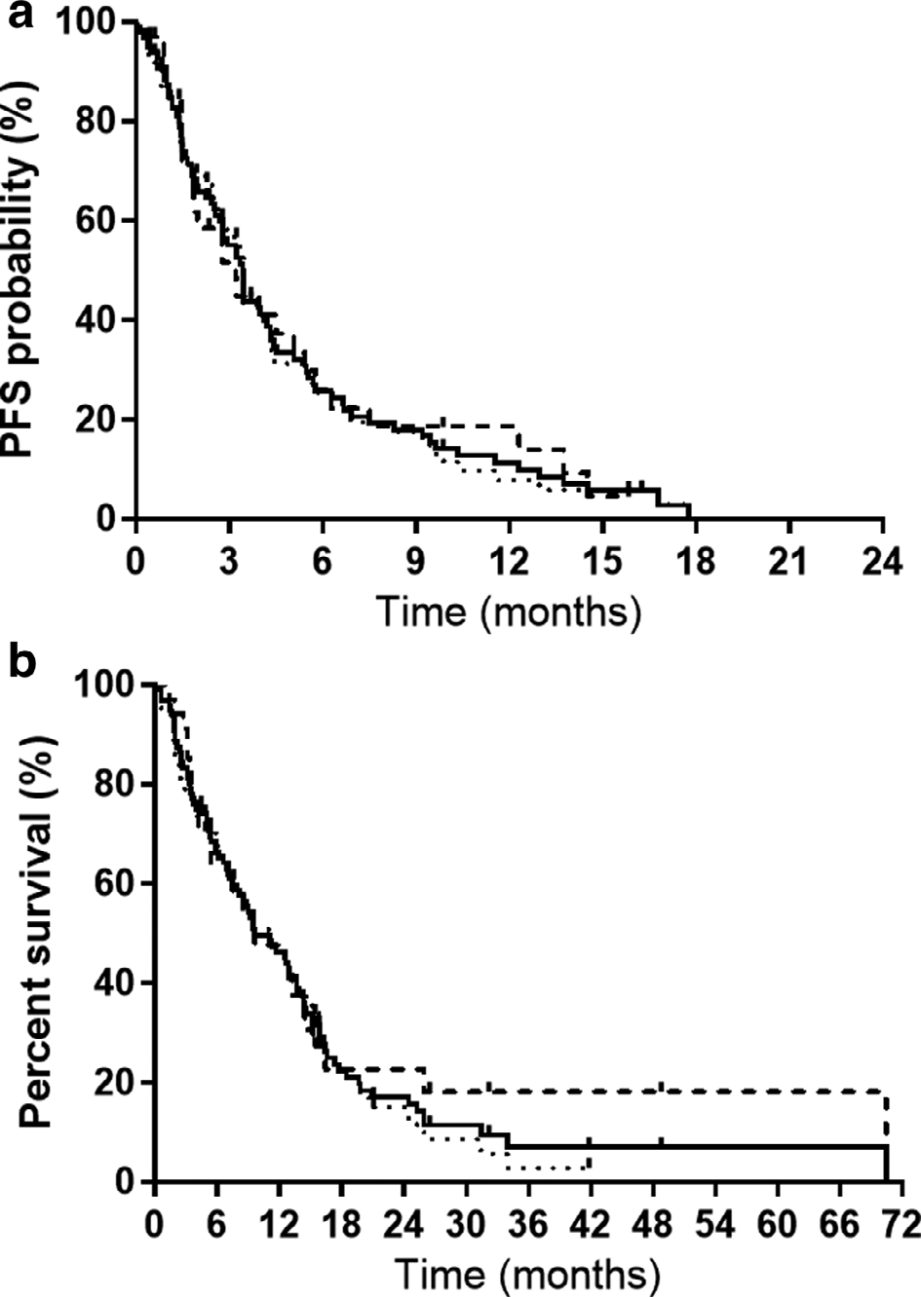
Kaplan–Meier analysis of (**a**) progression‐free survival (PFS); and (**b**) overall survival for the 96 patients in the study. (**a**) The median PFS for all the patients was 3.4 months, the median PFS associated with second‐line treatment was 3.1 months, and the median PFS associated with third‐ and subsequent‐line treatment was 3.4 months. 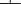 Total (*n* = 96), 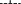 second‐line (*n* = 34), 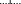 ≥third‐line (*n* = 62). (**b**) The median overall survival for all the patients was 9.6 months. The median overall survival associated with second‐line treatment was 9.6 months, and the median overall survival associated with third‐ and subsequent‐line treatment was 11.0 months. The median follow‐up time was 9.1 months. 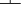
Total (*n* = 96), 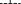
second‐line (*n* = 34), 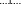
≥third‐line (*n* = 62).

Table 2 lists therapeutic responses for subsequent‐line S‐1 monotherapy. In brief, none of the patients achieved CR and 8, 34, and 41 patients met the criteria for PR, SD, and PD during the follow‐up period, respectively. The ORR and DCR were 8.3% (95% CI: 2.8–13.8) and 43.8% (95% CI: 33.8–53.6), respectively. Stratification based on age (75–79 vs. ≥80 years), PS score (0–1 vs. 2–4), and histological type (adenocarcinoma *vs*. squamous‐cell carcinoma) demonstrated no statistically significant differences between these groups regarding ORR or DCR. The stratification based on treatment line (second‐line vs. third‐ or later‐line) showed significant differences between these two groups for ORR (*P* = 0.04). The PFS and OS were 3.4 months (95% CI: 2.6–4.2) and 9.6 months (95% CI: 7.4–13.6), respectively (Fig 1a,b). Of the 96 patients, 81 (84.4%) died during the follow‐up period. The median PFS in the second‐line versus third‐ or later‐line therapy groups was 3.1 months (95% CI: 1.7–5.4) and 3.4 months (95% CI: 2.5–4.2), respectively. The median OS in the second‐line versus third‐ or later‐line therapy groups was 9.6 months (95% CI: 5.3–14.4) and 11.0 months (95% CI: 6.8–14.2), respectively. No statistically significant differences in PFS and OS were found between the two groups (Fig 1).

**Table 2:** Treatment response

Response	*N* = 96	(%)	Age 75–79/≥80 years	*P*‐value	PS 0–1/2–4	*P*‐value	Ad/Sq	*P*‐value	Second‐line/≥third‐line	*P*‐value
CR	0	0	0/0		0/0		0/0		0/0	
PR	8	8.3	6/2		8/0		4/4		0/8	
SD	34	35.4	28/6		32/2		17/13		13/21	
PD	41	42.7	28/13		35/6		23/15		16/25	
NE	13	13.5	8/5		13/0		9/3		5/8	
RR (%)	8.3	2.8–13.8^†^	8.6/7.7	0.99	9.1/0	0.99	7.5/11.4	0.71	0/12.9	**0.04**
DCR (%)	43.8	33.8–53.6^†^	48.6/30.8	0.21	45.5/25.0	0.15	39.6/48.6	0.81	38.2/46.8	0.49

Bold *P*‐values are statistically significant (*P* < 0.05).

Ad, adenocarcinoma; CR, complete response; DCR, disease control rate; NE, not evaluated; PD, progressive disease; PR, partial response; PS, performance status (score); RR, response rate; SD, stable disease; Sq, squamous cell carcinoma.

^†^ 95% confidence interval.

We compared the non‐PD (PR + SD) group (*n* = 42) with the PD group (*n* = 41) and, as shown in Table S2 (Online Resource 2), found no significant differences in any of the patients' characteristics. Furthermore, there were no significant differences in the characteristics of patients with a PFS <3 months versus those with a PFS >3 months, or in those with a PFS of <6 months versus those with a PFS >6 months (Table S3; Online Resource 3).

We further assessed multiple values for their prognostic value regarding PFS and OS (Table 3). Univariate analyses showed that the response other than PD to S‐1 monotherapy was significantly correlated with a longer PFS. Furthermore, the administration of S‐1 for four weeks followed by two weeks of rest and a response other than PD to S‐1 monotherapy were significantly correlated with a longer OS. Multivariate analysis showed that good PS (score: 0–1) at the start of S‐1 monotherapy (*P* = 0.04) and response to S‐1 treatment (*P* < 0.05) were independently correlated with improved PFS. The impact of S‐1 alone on the median PFS was significantly influenced by the response (the PFS among patients with non‐PD and PD status were 5.6 months and 1.8 months, respectively; log‐rank *P* < 0.0001). Multivariate analyses also demonstrated that patients with a status other than PD had a longer median OS than those with a PD status (14.5 months vs. 6.8 months; log‐rank *P* < 0.0001).

**Table 3:** Associations of clinical factors with PFS and OS

		Univariate analysis			Multivariate analysis				Univariate analysis			Multivariate analysis		
		PFS			PFS				OS			OS		
Factors	Median PFS (months)	HR	95% CI	*P*‐value	HR	95% CI	*P*‐value	Median OS (months)	HR	95% CI	*P*‐value	HR	95% CI	*P*‐value
Sex														
Male/female	3.4/3.4	0.91	0.56–1.51	0.7				9.2/13.2	1.17	0.73–1.92	0.51			
Age (years) at the start of S‐1														
75–79/≥80	3.4/3.4	0.92	0.56–1.57	0.76				12.4/8.0	0.84	0.52–1.40	0.49			
PS (ECOG) at the start of S‐1														
0–1/2–4	3.4/2.6	0.92	0.47–2.10	0.84	0.45	0.18–0.99	**0.04**	11.0/6.1	0.47	0.23–1.07	0.07	0.56	0.27–1.32	0.17
Smoking status														
Current or former/never	3.4/3.4	0.91	0.57–1.50	0.72				9.2/13.6	1.17	0.74–1.91	0.49			
Histology														
Ad/non‐ad	2.9/3.4	1.09	0.70–1.72	0.68	0.71	0.57–1.46	0.91	12.4/8.7	0.67	0.42–1.05	0.08	0.7	0.44–1.11	0.13
Treatment line														
2/≥3	3.1/3.4	0.95	0.58–1.50	0.83				9.6/11.0	0.85	0.52–1.36	0.52			
Driver mutation/translocation status														
Positive/negative or unknown	4.2/3.3	0.76	0.39–1.33	0.35				13.9/9.2	0.7	0.37–1.23	0.23			
Disease extent at diagnosis														
III–IV/postoperative recurrence	3.3/3.4	0.98	0.54–1.98	0.97				9.6/11.6	0.85	0.47–1.71	0.63			
Administration of S‐1														
2w1w/4w2w	3.4/3.4	1.1	0.68–1.80	0.68	1.07	0.64–1.80	0.77	8.7/15.2	1.97	1.19–3.28	**0.007**	1.63	0.97–2.78	0.06
Administration of ICIs														
Yes/No	2.7/3.4	1.24	0.72–2.03	0.41				12.8/9.4	0.71	0.40–1.20	0.21			
Response to S‐1														
Non‐PD/PD	5.6/1.8	0.26	0.16–0.42	**<0.0001**	0.2	0.11–0.35	**<0.0001**	14.5/6.8	0.48	0.30–0.78	**0.003**	0.49	0.30–0.80	**0.004**

Bold *P*‐values are statistically significant (*P* < 0.05).

2w1w, two weeks of S‐1 administration followed by one week of rest; 4w2w, four weeks of S‐1 administration followed by two weeks of rest; Ad, adenocarcinoma; CI, confidence interval; ECOG, Eastern Cooperative Oncology Group; HR, hazard ratio; ICI, immune checkpoint inhibitor; OS, overall survival; PD, progressive disease; PFS, progression‐free survival; PS, performance status.

### Feasibility and adverse event profiles

Table 4 lists AEs that occurred during S‐1 monotherapy, with the most common being anorexia (*n* = 35 [36.5%]; grade ≥ 3 in 9.4%) and anemia (*n* = 30 [31.3%]; grade ≥ 3 in 3.1%), followed by nausea (*n* = 24, 25.0%). A total of 22 patients (22.9%) discontinued treatment owing to AEs. Adverse events that resulted from discontinuation of treatment were as follows: grade 3 or grade 4 anorexia in seven patients, diarrhea in two patients, infection in one patient, and thrombocytopenia in one patient. Other significant adverse events were grade 3 (*n* = 1) infection and grade 4 (*n* = 1) bleeding. Treatment‐related death occurred in one patient (1.0%) due to drug‐induced pneumonitis; this patient was the only individual in the cohort to experience pneumonitis. In that case, emphysema was seen in the lung field, but no interstitial lung disease was found.

**Table 4:** Treatment‐related adverse events (*n* = 96)

	Any grade		Grade ≥ 3	
Adverse event	n	%	n	%
Led to discontinuation	22	22.9	12	12.5
Led to death	1	1	1	1
Hematologic toxicities				
Anemia	30	31.3	3	3.1
Platelet count decreased	18	18.7	2	2.1
Neutrophil count decreased	8	8.3	0	0
White blood cell decreased	6	6.3	0	0
Nonhematologic toxicities				
Anorexia	35	36.5	9	9.4
Nausea	24	25	3	3.1
Fatigue	18	18.7	1	1
Skin hyperpigmentation	10	10.4	0	0
Diarrhea	9	9.4	1	1
Mucositis oral	8	8.3	2	2.1
AST/ALT elevation	5	5.2	0	0
Watering eyes	5	5.2	0	0

Drug‐related adverse events occurring in 5% or more of patients are shown. One treatment‐related death was due to pneumonitis.

AST, aspartate aminotransferase; ALT, alanine aminotransferase.

### Treatment beyond disease progression

One patient was still receiving subsequent‐line S‐1 monotherapy by the end of the follow‐up period and had not experienced PD. Table 5 lists chemotherapeutic regimens delivered post‐recurrence to 43 of the patients following S‐1 monotherapy; the remaining 53 patients received palliative treatment without any further chemotherapy. Among patients treated beyond S‐1 monotherapy, the most common chemotherapeutic regimen was a cytotoxic agent; five received platinum‐based combination chemotherapy, but most received docetaxel, vinorelbine, or gemcitabine monotherapy. A total of 17 patients were administered EGFR‐TKIs as the subsequent‐line treatment post‐S‐1 monotherapy, including a first‐ or second‐generation EGFR‐TKI (gefitinib, erlotinib, and afatinib) re‐administration for those who were *EGFR* T790M‐negative (*n* = 12) and osimertinib for those who were T790M‐positive (*n* = 5). A total of 13 patients were administered immune‐checkpoint inhibitor (ICI) alone as the subsequent‐line treatment.

**Table 5:** Chemotherapeutic regimens administered upon disease progression following S‐1 monotherapy

	First‐subsequent regimen (*n* = 43)	Second‐subsequent regimen (*n* = 18)	Third‐subsequent regimen (*n* = 7)	Fourth‐subsequent regimen (*n* = 3)	Subsequent line regimens after S‐1 monotherapy (total number of patients)
Platinum combination	2	2	0	1	5
Docetaxel	4	2	2	1	9
Pemetrexed	4	0	1	0	5
Vinorelbine	6	2	0	0	8
Gemcitabine	5	2	0	0	7
S‐1 re‐challenge	0	2	0	0	2
Other cytotoxic drug monotherapy	3	1	1	0	5
EGFR‐TKIs					
Gefitinib/erlotinib/afatinib	7	3	1	1	12
Osimertinib	2	2	1	0	5
ICI monotherapy	10	2	1	0	13
Investigational agent	0	0	0	0	0
Chemoradiotherapy	0	0	0	0	0

EGFR‐TKIs, epidermal growth factor receptor tyrosine kinase inhibitors; ICI, immune checkpoint inhibitor.

*Of the 96 patients in the study, 53 received best supportive care immediately following S‐1 cessation and were not included in this table.

## Discussion

This study is the first to evaluate the effectiveness and tolerability of subsequent‐line S‐1 treatment alone in elderly patients (aged 75 years and older) in a real‐world setting. The results showed that subsequent‐line S‐1 monotherapy is likely safe and effective for this group of patients.

ICIs have become the preferred therapy for disease progression after front‐line platinum‐combination chemotherapy.^16^, ^17^, ^18^ Cytotoxic drugs (eg, pemetrexed, S‐1, and docetaxel with or without ramucirumab) are also a standard treatment for patients with previously treated NSCLC,^10^, ^19^, ^20^, ^21^ and are approved monotherapies for subsequent‐line settings. Both the response rate and toxicity associated with S‐1 monotherapy are similar to those associated with other monotherapeutic cytotoxic agents in elderly patients pretreated for NSCLC.^10^, ^19^, ^20^, ^21^ In our study, the ORR following subsequent‐line S‐1 monotherapy was 8.3%, which is equivalent to that found in the previously performed EAST‐LC prospective phase III study (*n* = 577, S‐1 group; ORR = 8.3%).^10^ Subsequent‐line S‐1 monotherapy was also associated with a PFS of 3.4 months among our patients, which is somewhat longer than that observed in the EAST‐LC study (2.86 months; 95% CI: 2.73–3.12).^10^ Therefore, S‐1 monotherapy may be appropriate for elderly patients with previously treated NSCLC.

There were no statistically significant differences in the characteristics of patients with PD and those with PR or SD, and there significant differences between patients with a PFS <3 months versus those with a PFS ≥3 months or patients with a PFS <6 months versus those with a PFS ≥6 months. However, the number of patients in this investigation might be too small for sufficient statistical power.

Patient cohorts that receive a survival advantage from subsequent‐line S‐1 monotherapy have not yet been identified. The multivariate analyses conducted in the current investigation showed that treatment response (non‐PD vs. PD) was an independent predictor of PFS and OS. Patients who attained disease control were more likely to experience greater PFS, which in turn was correlated with a longer OS. Furthermore, compared with patients in our cohort with good PS scores (0–1), those with scores of 2–3 at the commencement of S‐1 monotherapy experienced a shorter median PFS and OS. It has previously been suggested that, at the patient level, the number of chemotherapeutic regimens received beyond disease progression with front‐line treatment is independently associated with post‐progression survival,^22^which could be prolonged in patients who are able to continue treatment (thereby also extending OS). Our results support this notion, given that subsequent‐line S‐1 monotherapy in patients with a controlled disease was associated with favorable prognosis.

Elderly patients generally have more complications and lower organ functions than younger patients; therefore, treatment‐related toxicities among elderly patients are a notable concern. Onset and severity of AEs were similar to those found in the EAST‐LC study.^10^ In our study, AEs were predictably correlated with the subsequent‐line S‐1 treatment, and most were low‐grade. Except for anorexia (9.4%), treatment‐related toxicities higher than grade 3 were found in less than 5% of patients. Treatment‐related death occurred in one patient (1.0%); however, 22 patients (22.9%) discontinued treatment due to AEs. In comparison, treatment discontinuation owing to AEs in the EAST‐LC study occurred in 49 of 576 patients (8.5%) in the S‐1 group.^10^ The discrepancy between these results demonstrates the well‐known limitations of clinical studies in evaluating pharmacological agent safeness and emphasize the demand for clinical practice situation.^23^ Notwithstanding, the safety of S‐1 alone found in the present investigation was similar to that reported in the EAST‐LC trial,^10^ and the rate of cytotoxic drug‐associated toxicities were better than those previously reported among elderly patients.^24^, ^25^ In a phase III study performed in Japan that assessed patients receiving vinorelbine and docetaxel, grade 3 and 4 neutropenia were reported in 69.3% and 82.9% of the patients, respectively.^25^ Even though the current study included patients aged 75 years and older in a subsequent‐line setting and the Japanese study enrolled patients aged 70 years and older in a first‐line setting, the occurrences of hematological and nonhematologic AEs were lower in the current study. Additionally, both the hematological and nonhematologic AEs in this study were manageable and controlled regardless of severity. This indicates that the AEs related to subsequent‐line S‐1 monotherapy among elderly patients with NSCLC are low‐grade, suggesting that this chemotherapeutic regimen is suitable and tolerable.

Although the standard chemotherapy administered to most patients with advanced or metastatic NSCLC is platinum‐combination chemotherapy containing new cytotoxic drugs such as paclitaxel, docetaxel, gemcitabine, and vinorelbine, the efficacy and safety of these drugs in elderly patients remain unclear.^26^, ^27^ Hence, there are no standard post‐chemotherapeutic regimens for such patients, and the influence of later‐line treatments on the OS of elderly patients with NSCLC who are administered S‐1 monotherapy remains unknown. The currently available treatment options for such patients include platinum‐ or nonplatinum‐base combination treatment, monotherapy with a third‐generation agent, and best supportive care.^28^ Approximately half of the patients in this study were treated with platinum‐based combination chemotherapy prior to S‐1 monotherapy (first‐line, *n* = 40; second‐line, *n* = 6; and third‐line, *n* = 5). Regarding the number of following treatments, 44.7% of the patients (43 of 96) were administered subsequent‐line treatment beyond S‐1 monotherapy (Table 5). The efficacy and safety of ICIs have been demonstrated to be favorable in previously treated elderly patients with NSCLC. Yamaguchi *et al*. reported that subsequent‐line ICI monotherapy was useful, feasible, and resulted in outcomes similar to those observed in the present study, although their study included only elderly patients.^29^ The optimal sequence of administration of cytotoxic drugs and ICIs is not yet clear; however, administering S‐1 monotherapy sometime after the first‐line treatment may be preferable for elderly patients with NSCLC.

There are several limitations of the current study. First, both the administration of S‐1 alone as subsequent‐line chemotherapy and the use of front‐line chemotherapy were decided by the attending physician. Furthermore, originally planned administration of S‐1 alone may have been omitted or delayed at the discretion of the treating physician. Second, S‐1 was administered beyond the second‐ or third‐line treatment, and thus the treatment line and S‐1 administration schedule were not uniform. To minimize the influence of these implicit origins of bias, all consecutive patients who received S‐1 monotherapy at our institutions were included in our investigation, and their medical records were wholly evaluated. Third, the sample size of our study was relatively modest. Fourth, our study was retrospective. Patient selection and the evaluation of the imaging schedule is important. Further investigations that involve direct comparisons with other studies should be very carefully performed and reported. Fifth, our results suggest that tumor response was a prognostic factor for PFS and OS in this analysis. However, we did not examine the predictive ability.

In conclusion, subsequent‐line S‐1 monotherapy is probably safe for elderly patients with previously treated NSCLC, and the outcomes observed in our study population were similar to those observed in studies that included nonelderly patients. Therefore, S‐1 monotherapy could be one of the treatment choices for elderly patients with previously treated NSCLC.

## Disclosure

None of the authors have any financial or personal relationships with people or organizations that could inappropriately influence this work.

## Supporting information


**Appendix S1.** Supporting information
