# Supplementary material for: Efficacy and safety of S‐1 monotherapy in previously treated elderly patients (aged ≥75 years) with non‐small cell lung cancer: A retrospective analysis
Source: Thorac Cancer. 2020 Aug 26;11(10):2867–76. doi: 10.1111/1759-7714.13622 (PMC7529563; doi:10.1111/1759-7714.13622)
Supplement: Supplementary file 1 — Appendix S1. Supporting information [file TCA-11-2867-s001.docx]

**Thoracic Cancer**

# Efficacy and safety of S-1 monotherapy in previously treated elderly patients (aged ≥75 years) with non-small cell lung cancer: A retrospective analysis

Hisao Imai, Hiroyuki Minemura, Takayuki Kishikawa, Yutaka Yamada, Kensuke Suzuki, Yukihiro Umeda, Satoshi Wasamoto, Norimitsu Kasahara, Shinichi Ishihara, Ou Yamaguchi, Ichiro Naruse, Junji Uchino, Keita Mori, Kenya Kanazawa, Yoko Shibata, Takashi Kasai, Takayuki Kaburagi, Kyoichi Kaira, Koichi Minato

**Corresponding author**

Hisao Imai, M.D., Ph.D., Division of Respiratory Medicine, Gunma Prefectural Cancer Center, 617-1, Takahayashinishi, Ota, Gunma 373-8550, Japan. Tel: +81-276-38-0771, Fax: +81-276-38-0614, E-mail: m06701014@gunma-u.ac.jp

Table S1. Chemotherapeutic regimens administered prior to S-1 monotherapy

|  | First-line (n = 96) | Second-line (n = 62) | Third-line (n = 35) | ≥Fourth-line (n = 20*) |
| --- | --- | --- | --- | --- |
| Platinum combination | 40 | 6 | 5 | 1 |
| Platinum combination + ICIs | 0 | 0 | 0 | 0 |
| Docetaxel | 22 | 14 | 10 | 2 |
| Pemetrexed | 5 | 5 | 4 | 1 |
| Vinorelbine | 4 | 7 | 2 | 2 |
| Gemcitabine | 0 | 2 | 5 | 5 |
| Other cytotoxic drug monotherapy | 1 | 6 | 0 | 2 |
| Non-platinum combination | 2 | 2 | 1 | 0 |
| EGFR-TKIs |  |  |  |  |
| Gefitinib/erlotinib/afatinib | 14 | 11 | 5 | 6 |
| Osimertinib | 0 | 1 | 0 | 1 |
| ICI monotherapy | 3 | 8 | 3 | 0 |
| Chemoradiotherapy | 5 | 0 | 0 | 0 |

*Total number of patients

ICIs: immune checkpoint inhibitors; EGFR-TKIs: epidermal growth factor receptor tyrosine kinase inhibitors

Table S2. Comparison of the non-PD (PR + SD) group (n = 42) with the PD group (n = 41)

|  | **n = 83*** | |  |
| --- | --- | --- | --- |
| **Factors** | **PR+SD (n = 42)** | **PD (n = 41)** | ***p*-value**** |
| Sex |  |  |  |
| Male | 31 | 30 | 0.99 |
| Female | 11 | 11 |  |
| Age (years) at the start of S-1 |  |  |  |
| 75–79 | 34 | 28 | 0.21 |
| ≥80 | 8 | 13 |  |
| Performance status (ECOG-PS) at the start of S-1 |  |  |  |
| 0–1 | 40 | 35 | 0.15 |
| 2–4 | 2 | 6 |  |
| Smoking status |  |  |  |
| Current or former | 32 | 26 | 0.23 |
| Never | 10 | 15 |  |
| Histology |  |  |  |
| Ad | 21 | 23 | 0.66 |
| Non-ad | 21 | 18 |  |
| Treatment line |  |  |  |
| 2nd | 13 | 16 | 0.49 |
| ≥3rd | 29 | 25 |  |
| Driver mutation/translocation status |  |  |  |
| Positive | 6 | 9 | 0.4 |
| Negative or unknown | 36 | 32 |  |
| Disease extent at diagnosis |  |  |  |
| III–IV | 36 | 37 | 0.73 |
| Postoperative recurrence | 6 | 4 |  |
| Administration of S-1*** |  |  |  |
| 2w1w | 19 | 17 | 0.99 |
| 4w2w | 17 | 17 |  |
| Administration of ICIs |  |  |  |
| Yes | 9 | 12 | 0.45 |
| No | 33 | 29 |  |

*Excluding 13 patients from the study who were not evaluated

**Fisher’s exact test

***Included 2w1w and 4w2w

PR: partial response; SD: stable disease; PD: progressive disease; ECOG: Eastern Cooperative Oncology Group; PS: performance status; Ad: adenocarcinoma; 2w1w: 2 weeks of S-1 administration followed by 1 week of rest; 4w2w: 4 weeks of S-1 administration followed by 2 weeks of rest; ICI: immune checkpoint inhibitor

Table S3. Comparison of patients who achieved PFS of <3 vs. ≥3 months and <6 vs. ≥6 months

| **Factors** | **PFS <3 months (n = 51)** | **PFS ≥3 months (n = 45)** | ***p*-value*** | **PFS <6 months (n = 76)** | **PFS ≥6 months (n = 20)** | ***p*-value*** |
| --- | --- | --- | --- | --- | --- | --- |
| Sex |  |  |  |  |  |  |
| Male | 35 | 33 | 0.65 | 52 | 16 | 0.41 |
| Female | 16 | 12 |  | 24 | 4 |  |
| Age (years) at the start of S-1 |  |  |  |  |  |  |
| 75–79 | 36 | 34 | 0.64 | 55 | 15 | 0.99 |
| ≥80 | 15 | 11 |  | 21 | 5 |  |
| ECOG PS score at the start of S-1 |  |  |  |  |  |  |
| 0–1 | 47 | 41 | 0.99 | 70 | 18 | 0.67 |
| 2–4 | 4 | 4 |  | 6 | 2 |  |
| Smoking status |  |  |  |  |  |  |
| Current or former | 34 | 33 | 0.51 | 52 | 15 | 0.78 |
| Never | 17 | 12 |  | 24 | 5 |  |
| Histology |  |  |  |  |  |  |
| Ad | 31 | 22 | 0.3 | 43 | 10 | 0.62 |
| Non-ad | 20 | 23 |  | 33 | 10 |  |
| Treatment line |  |  |  |  |  |  |
| 2nd line | 19 | 15 | 0.83 | 27 | 7 | 0.99 |
| ≥3rd line | 32 | 30 |  | 49 | 13 |  |
| Driver mutation/translocation status |  |  |  |  |  |  |
| Positive | 8 | 8 | 0.79 | 11 | 5 | 0.31 |
| Negative or unknown | 43 | 37 |  | 65 | 15 |  |
| Disease extent at diagnosis |  |  |  |  |  |  |
| III–IV | 47 | 38 | 0.33 | 68 | 17 | 0.69 |
| Postoperative recurrence | 4 | 7 |  | 8 | 3 |  |
| Administration of S-1** |  |  |  |  |  |  |
| 2w1w | 21 | 22 | 0.82 | 32 | 11 | 0.59 |
| 4w2w | 19 | 17 |  | 29 | 7 |  |
| Administration of ICIs |  |  |  |  |  |  |
| Yes | 14 | 9 | 0.47 | 19 | 4 | 0.77 |
| No | 37 | 36 |  | 57 | 16 |  |

*Fisher’s exact test

**Included 2w1w and 4w2w

PFS: progression-free survival; ECOG: Eastern Cooperative Oncology Group; PS: performance status; Ad: adenocarcinoma; 2w1w: 2 weeks of S-1 administration followed by 1 week of rest; 4w2w: 4 weeks of S-1 administration followed by 2 weeks of rest; ICI: immune checkpoint inhibitor
